# Copper Nanoparticles Synthesized by Chemical Reduction with Medical Applications

**DOI:** 10.3390/ijms26041628

**Published:** 2025-02-14

**Authors:** Alexandra Pricop, Adina Negrea, Bogdan Pascu, Nicoleta Sorina Nemeş, Mihaela Ciopec, Petru Negrea, Cătălin Ianăşi, Paula Svera, Delia Muntean, Alexandra Ivan, Iustina Mirabela Cristea

**Affiliations:** 1 Faculty of Chemical Engineering, Biotechnologies and Environmental Protection, Politehnica University Timişoara, Victoriei Square, no. 2, 300006 Timişoara, Romania; maria.pricop@student.upt.ro (A.P.); adina.negrea@upt.ro (A.N.); mihaela.ciopec@upt.ro (M.C.); petru.negrea@upt.ro (P.N.); 2 Research Institute for Renewable Energies—ICER, Politehnica University Timişoara, Gavril Musicescu Street, no. 138, 300774 Timisoara, Romania; 3 Coriolan Drăgulescu’ Institute of Chemistry, Bv. Mihai Viteazul, No. 24, 300223 Timisoara, Romania; ianasic@acad-icht.tm.edu.ro; 4 INCEMC—National Institute for Research and Development in Electrochemistry and Condensed Matter-Timisoara, No. 144 Dr. A. Paunescu Podeanu Street, 300569 Timisoara, Romania; paulasvera@gmail.com; 5 Multidisciplinary Research Centre on Antimicrobial Resistance, Department of Microbiology, University of Medicine and Pharmacy “Victor Babes”, Eftimie Murgu Sq. No. 2, 300041 Timişoara, Romania; muntean.delia@umft.ro; 6 Center of Immuno-Physiology and Biotechnologies (CIFBIOTEH), University of Medicine and Pharmacy “Victor Babes”, Eftimie Murgu Sq. No. 2, 300041 Timisoara, Romania; ivan.alexandra@umft.ro; 7 OncoGen Centre, Clinical County Hospital “Pius Branzeu”, Blvd. Liviu Rebreanu 156, 300723 Timisoara, Romania; mirabela.cristea@oncogen.ro

**Keywords:** copper nanoparticles, chemical reduction, cytotoxicity effect, antibacterial activity

## Abstract

Copper nanoparticles (CuNPs) have attracted attention due to their low cost and high specific surface area. In this work, a simple and inexpensive two-step synthesis method was proposed to prepare highly stable and well-dispersed spherical CuNPs in solution with a particle size of approximately 37 nm. Synthesis of CuNPs was carried on in the presence of complexing agent trisodium citrate (TSC), while for the chemical reduction step, sodium borohydride (NaBH_4_) was used. Taking into account the potential of this type of nanoparticles, their synthesis and characterization represent a current and relevant topic in the field. The ability to control the size, shape and properties of CuNPs by adjusting the synthesis parameters (pH, precursor:stabilizer:reductant ratio, homogenization time, temperature) offers extraordinary flexibility in the development of these materials. The combination of characterization techniques such as SEM, EDX, UV–Vis, Raman, FT-IR and AFM provides a thorough understanding of the structure and properties of CuNPs, allowing the modulation of the properties of the obtained nanoparticles in the desired direction. Based on the studies, the copper reduction mechanism was proposed. For the theoretical verification of the size of the experimentally obtained spherical CuNPs, Mie theory was applied. A stability study of the synthesized CuNPs in optimal conditions was performed using UV–Vis analysis at specific time intervals (1, 3, 30 and 60 days), the sample being kept in the dark, inside a drawer at 25 °C. The CuNPs obtained after setting the optimal synthesis parameters (Cu(II):TSC:${BH}_{4}^{+}$ = 1:1:0.2, pH = 5, homogenization time 60 min and temperature 25 °C) were then tested to highlight their antibacterial effect on some reference bacterial strains. The obtained CuNPs demonstrated very good antimicrobial efficacy compared to traditional antimicrobials, for both Gram-negative and Gram-positive bacteria. This may reduce the development of antimicrobial resistance, an urgent medical issue. After evaluating the cytotoxic effects of CuNPs on the SKBR3 cancer cell line, a significant decrease in cell proliferation was observed at the 0.5 mg/mL concentration, with a reduction of 89% after 60 h of cultivation. Higher concentrations of CuNPs induced a more rapid cytotoxic effect, leading to an accelerated decline in cell viability.

## 1. Introduction

Nanotechnology has become a central subject in modern science and industry, transforming fields such as medicine [1,2], electronics [3], energy [4] and environmental science [5] by producing materials with new physical, chemical and biological properties, on a nanometric scale [6,7]. A key component of nanotechnology is the creation of nanostructured materials with sizes ranging from 1 to 100 nm. Depending on the application, nanoparticles can be made of metals, oxides, carbon or polymers. Nanoparticles can be categorized mostly according to their source or precursor, form and material composition [8]. In the last few years, considerable interest has been shown in metal nanoparticles due to their special properties and potential applications in various fields [9,10]. Metallic nanoparticles have special electrical, mechanical, magnetic and chemical capabilities because of their small size and huge specific surface area [11]. These factors have led to the use of metal nanoparticles in a wide range of industries, including electronics, optoelectronics, photonics, biosensors, catalysis and medicine [12,13,14,15,16,17].

Because of their functional diversity and cost-effectiveness, CuNPs are a significant class of metal nanoparticles that have attracted considerable attention due to their properties [18,19]. Because of their superior electrical conductivity, catalytic qualities and low cost compared to silver and gold material, the synthesis of copper nanoparticles has generated much attention over the years [20,21,22,23]. CuNPs can be synthesized using a range of physical, chemical and biological techniques [24,25], each of them with specific advantages and disadvantages. Physically synthesized nanoparticles by methods like mechanical ball milling, physical vapor deposition or gas evaporation have the advantage of uniform distribution and lack solvent contamination, which sets them apart from chemically synthesized ones, such as liquid-phase chemical reduction [26,27], chemical deposition [28] and electrochemical and hydrothermal methods [29,30]. However, they have disadvantages such as slow unfolding over time and high energy consumption at high operating temperature [11,31,32].

Biological methods are increasingly used due to the low cost and non-toxic and environmentally friendly nature of the reducing agents [33,34]. Nanoparticles obtained by biological methods have excellent antimicrobial, anticancer, antidiabetic, anti-inflammatory and antioxidant activities [35]. In terms of chemical methods for obtaining CuNPs, there are a multitude of techniques, including chemical reduction, microwave reduction, sonochemical reduction, electrochemical reduction, photochemical reduction, microemulsion method, hydrothermal method, sol-gel method and thermal decomposition method [33]. Chemical reduction stands out among these techniques due to its selectivity and ability to control the size of the final nanoparticles, two crucial factors for adjusting the biological response of nanoparticles.

Copper sulfate (CuSO_4_), copper chloride (CuCl_2_) and copper acetate (Cu(CH_3_COO)_2_) are some of the most commonly utilized precursors for CuNPs. These precursors are reduced under carefully controlled circumstances to produce the desired nanoparticles [36,37]. For the reduction of Cu(II), the most commonly used reducing agents are sodium borohydride [38], polyols [39,40], hydrazine [41,42], ascorbic acid [43,44], glucose [45,46] and ethylene glycol [45,47], among others.

Therefore, the properties of CuNPs are influenced by the synthesis method used, having a crucial role in their applications, especially in biomedical research. The size of nanoparticles, which can be adjusted during synthesis, is an essential aspect, as it allows for the specific adjustment of optical, catalytic, electrical and biological properties [48].

In these terms, the study’s objective is to create CuNPs through chemical reduction with sodium borohydride, applying simple and effective methods for CuNPs synthesis which can be used for different medical applications. For that reason, the material’s antibacterial properties and cytotoxic effect were evaluated.

Infections caused by resistant pathogens are a significant cause of severe infections worldwide [49,50,51,52]. These pathogens are increasingly difficult to combat due to the resistance acquired over time to the antibiotics used to treat these infections, leading to various forms of cancer in the case of prolonged chronic infections. Therefore, any newly synthesized material that demonstrates antimicrobial properties deserves to be studied in detail, in order to be subsequently used in various medical applications. In this context, it is possible to analyze how CuNPs interact with bacterial strains to combat bacterial proliferation.

On the other hand, the cytotoxic role of certain substances or agents means the ability of these to cause cell death [8,53,54]. This effect can be seen in a variety of situations, including (i) cancer treatment, where a variety of chemotherapies and antimicrobial medications are intended to have a cytotoxic effect on tumor cells, thereby limiting their proliferation; (ii) viral infections, where certain viruses have the ability to cause host cell death as part of their replication cycle, thereby compromising tissue health; and (iii) immune reactions, where specific immune cells, like cytotoxic T lymphocytes, have the function of eliminating diseased or aberrant cells.

The literature reports that CuNPs are more cytotoxic to human cells than other metal oxide nanoparticles [55]. Therefore, CuNPs can be used in the treatment of several types of cancer, including liver cancer [56], lung cancer [57] mammalian cancer [58,59], cervical cancer [60] and pancreatic cancer [61].

It must be noted that copper is an essential trace element vital for human health, serving as a cofactor in processes like blood clotting, hormone maturation and cellular energy production [62,63,64]. Its redox properties enable participation in critical biochemical reactions, though they can also lead to toxicity [65]. On the other hand, copper plays a significant role in tumor cell death through mechanisms such as cuproptosis, which disrupts mitochondrial respiration, and paraptosis, characterized by endoplasmic reticulum stress and cytoplasmic vacuolization [66]. Copper and its complexes exhibit anticancer effects by inducing oxidative stress, inhibiting angiogenesis, arresting the cell cycle, and regulating energy metabolism [67,68,69]. Due to their cytotoxic potential against cancer cells, CuNPs have recently become a focus of extensive research [70]. These nanoparticles can generate ROS, causing membrane lipid peroxidation, DNA damage and cell proliferation inhibition, offering innovative strategies for anticancer drug development [71]. Comparative studies on CuNPs and ionic copper cytotoxicity indicate that the toxicity of CuNPs is mainly due to particulate copper rather than released ions [72,73].

In this sense, the obtained CuNPs open the way to antitumor treatments, specific to many types of cancer, and the detailed study of these nanoparticles may allow us to obtain key parameters that are necessary for the manifestation of antitumor action for certain types of cancer cells.

## 2. Results and Discussion

### 2.1. Establishing the Optimal Parameters for the Synthesis of CuNPs by Chemical Reduction

#### 2.1.1. Determining the Amount of Complexing Agent in the Synthesis of CuNPs

**Figure 1:**
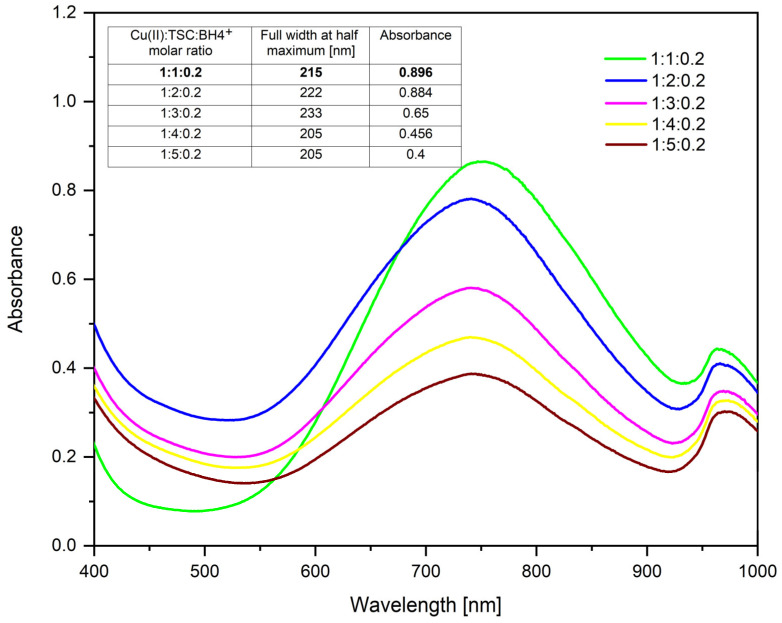
TSC influence (1M CuCl_2_ · 2H_2_O; 0.2 g NaBH_4_; 60 min homogenous time; pH = 5; 25 °C temperature).

Determining the optimal amount of complexing agent is an important aspect in many chemical processes, from chemical analysis to materials synthesis. By varying the amount of TSC and keeping the amounts of precursor and reducing agent constant, the following UV–Vis spectra were obtained (Figure 1).

**Figure 2:**
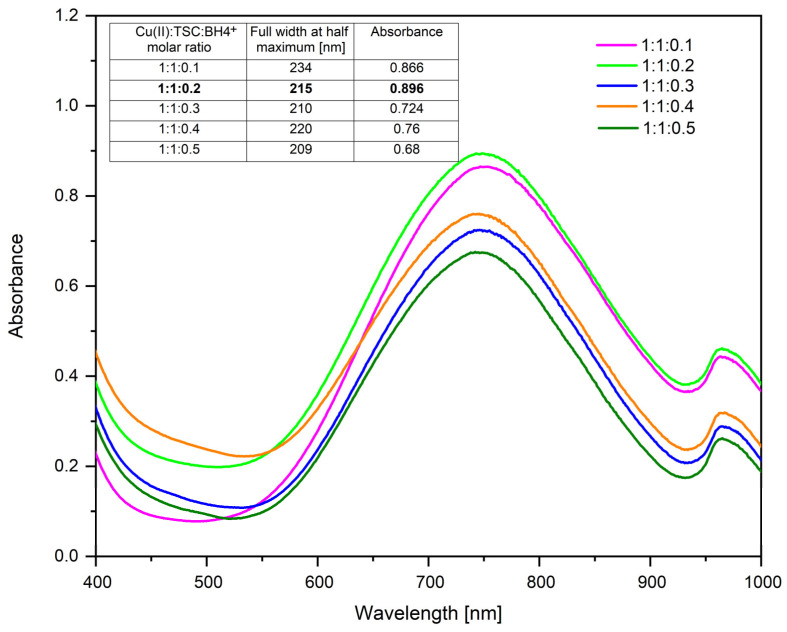
NaBH_4_ influence (1M CuCl_2_ · 2H_2_O; 1M TSC; 60 min homogenous time; pH = 5; 25 °C temperature).

According to Figure 1, the surface plasmon resonance existing at wavelength λ = ~746 nm indicates the presence of CuNPs [74]. Using Gaussian fits for the molar ratios Cu(II):TSC:${BH}_{4}^{+}$ used in the synthesis of CuNPs and presented in Figure 2, the full width at half maximum (FWHM) values indicate that the ratios Cu(II):TSC:${BH}_{4}^{+}$ = 1:4:0.2 and Cu(II):TSC:${BH}_{4}^{+}$ = 1:5:0.2 present a lower FWHM value. The absorbance of the material obtained by the synthesis at the molar ratio Cu(II):TSC:${BH}_{4}^{+}$ = 1:1:0.2 is clearly higher, indicating the highest concentration of CuNPs [75]. An increase in the intensity of the surface plasmon resonance band (SPR) indicates the formation and increase in the number of nanoparticles. Furthermore, the SPR band width can be correlated with the nanoparticle size distribution [76]. A wider band indicates a wider size distribution. Based on these considerations, the optimum molar ratio is Cu(II):TSC:${BH}_{4}^{+}$ = 1:1:0.2.

#### 2.1.2. Determining the Amount of Reducing Agent, NaBH_4_, in the Synthesis of CuNPs

Chemical reduction with sodium borohydride is one of the most widely used methods, due to the rapid reduction rate and relatively easy control of particle size. Determining the optimal amount of NaBH_4_ in the synthesis of CuNPs is a crucial aspect for obtaining nanoparticles with desired sizes, shapes and properties.

The rate at which copper ions are reduced to metallic copper is directly influenced by the concentration of NaBH_4_. An excessive amount might cause reduction to occur excessively quickly, which would encourage the creation of large, clumped nanoparticles.

Figure 2 shows the UV–Vis spectra for the molar ratios Cu(II):TSC:${BH}_{4}^{+}$, varying the amount of NaBH_4_ and keeping the amounts of precursor and complexing agent constant. The FWHM for the studied molar ratios is also established.

From the resulting spectra, it can be observed that at the molar ratio Cu(II):TSC:${BH}_{4}^{+}$ = 1:1:0.2 is the highest absorbance, which is correlated with the highest concentration of CuNPs [75]. The full width at half maximum and the absorbance values are also presented in Figure 2.

From the results obtained, it is observed that the values of full width at half maximum are relatively similar, indicating that there are no major differences in terms of the monodispersity of the colloidal system. However, there are differences in terms of absorbance, the optimal one being 0.896, which belongs to the molar ratio Cu(II):TSC:${BH}_{4}^{+}$ = 1:1:0.2, which is considered the optimal synthesis ratio.

#### 2.1.3. Establishing the Role of Optimal pH in the Synthesis of CuNPs

**Figure 3:**
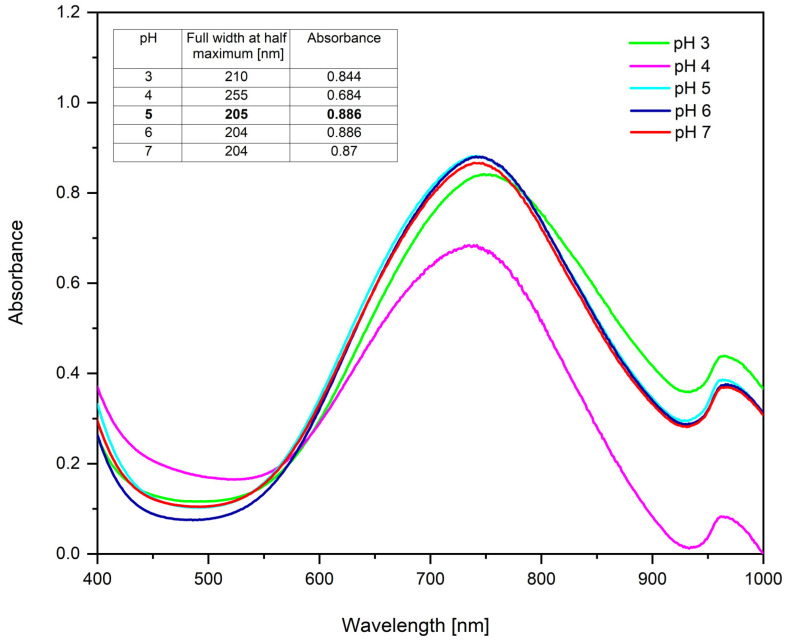
pH influence for optimal molar ratio Cu(II):TSC:${BH}_{4}^{+}$ = 1:1:0.2; 60 min homogenous time; 25 °C temperature.

pH influences the stability of ${BH}_{4}^{+}$ and can affect the rate of the precursor’s reduction. To highlight the influence of pH, UV–Vis spectra were obtained at different pH values at the molar ratio Cu(II):TSC:${BH}_{4}^{+}$ = 1:1:0.2, which was considered the optimal molar ratio (Figure 3).

From the spectra obtained, it can be observed that the optimal pH is between 5 and 6, because the spectra’s absorbance is higher, indicating the highest concentration of CuNPs [77,78,79]. Full width at half maximum was also calculated.

From the full width at half maximum analysis, it can be observed that all samples have similar values, indicating that there are no major changes in the monodispersity of the colloidal system. However, at pH < 5 and pH > 5, after 24 h, a precipitate appears, indicating that the nanoparticles are not stable. Therefore, subsequent studies were carried out at pH = 5.

#### 2.1.4. Establishing the Role of Homogenization Time in the Synthesis of CuNPs

**Figure 4:**
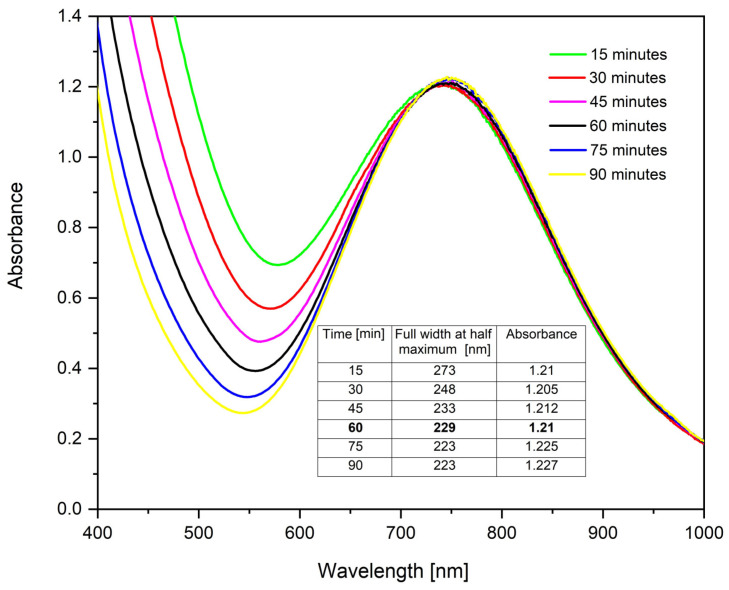
Homogenization time influence for the molar ratio Cu(II):TSC:${BH}_{4}^{+}$ = 1:1:0.2; 25 °C temperature and pH = 5.

Time-dependent UV–Vis measurements provide valuable information about the kinetics of the CuNPs forming and the evolution of their optical properties. Figure 4 shows the UV–Vis spectra for the samples synthesized at different homogenization times, maintaining the molar ratio Cu(II):TSC:${BH}_{4}^{+}$ = 1:1:0.2 and pH = 5.

It can be observed that the synthesis does not take place instantly and is completed after 90 min. Moreover, for more detailed study, full width at half maximum determinations were performed. From the obtained results, it is observed that with the increase in the homogenization time, full width at half maximum decreases to λ = 223 nm, the absorbance increases and implicitly the CuNP concentration increases, which indicates that the monodispersity of the colloidal system increases [80]. As no major changes in absorbance or full width at half maximum occur between 60 and 90 min, a homogenization time of 60 min was considered sufficient for subsequent studies.

#### 2.1.5. Establishing the Role of Temperature in the Synthesis of CuNPs

**Figure 5:**
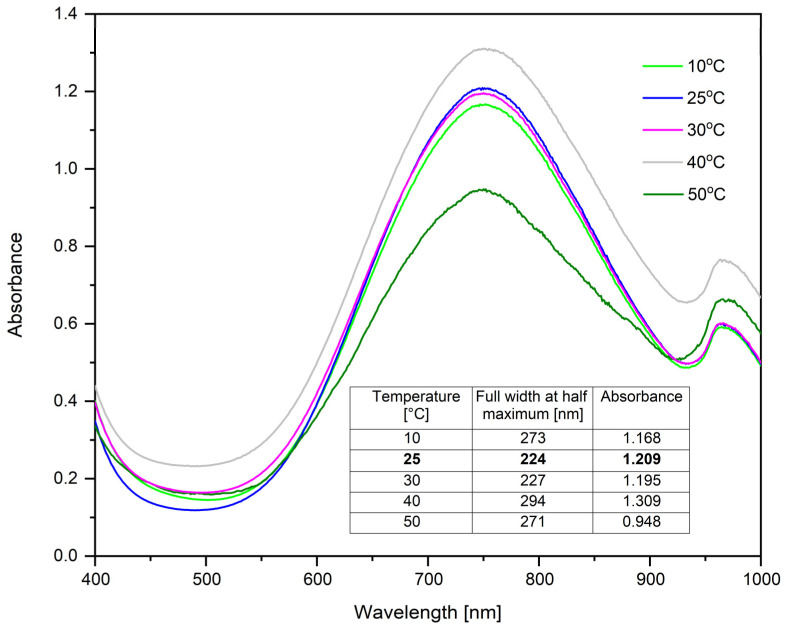
Temperature influence for the molar ratio Cu(II):TSC:${BH}_{4}^{+}$ = 1:1:0.2, pH = 5 and homogenization time 60 min.

To investigate the influence of temperature on the synthesis of CuNPs, UV–Vis spectra were obtained at different temperature values (Figure 5), maintaining constant the molar ratio Cu(II):TSC:${BH}_{4}^{+}$ = 1:1:0.2, pH = 5 and homogenization time of 60 min.

The optimal temperature for this synthesis is between 10 and 30 °C, as can be seen in Figure 5. Although an increase in absorbance can be observed at 40 °C, this sample is unstable, because after 5 h, a precipitate was observed. The full width at half maximum determination was also performed.

Regarding the sample synthesized at 10 °C, the full width at half maximum is higher compared to the samples synthesized at 25 °C and 30 °C. On the other hand, the absorbance is lower, which indicates that the monodispersity of the colloidal system is better for the samples synthesized at 25 °C and 30 °C. The concentration of CuNPs is higher in the case of the samples synthesized at 25 °C and 30 °C. For economic reasons, the optimal synthesis temperature was considered to be 25 °C.

#### 2.1.6. Study on the Stability of CuNPs over Time

**Figure 6:**
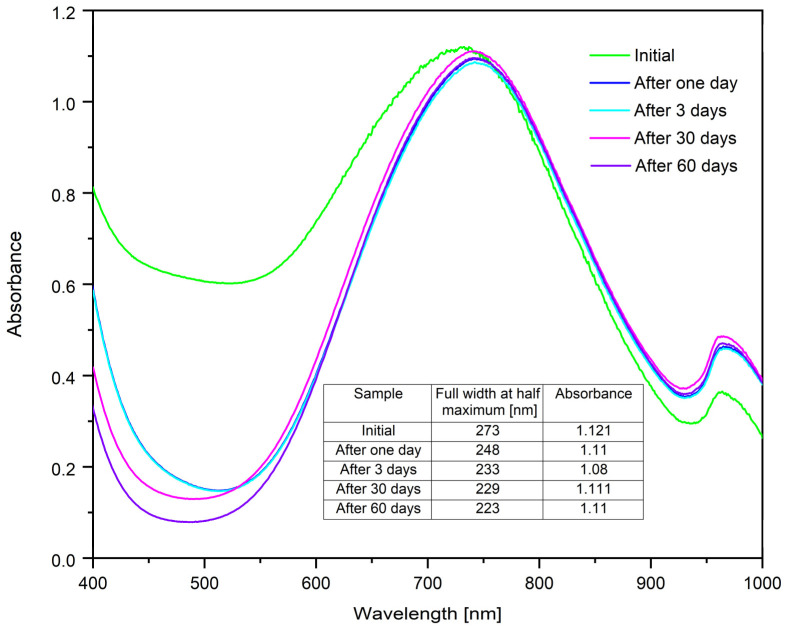
Stability study for the sample synthesized under optimal conditions: molar ratio Cu(II):TSC:${BH}_{4}^{+}$ = 1:1:0.2, pH = 5, homogenization time 60 min and temperature 25 °C.

To highlight the stability of CuNPs, UV–Vis spectra were obtained at different time intervals (Figure 6) for the sample synthesized under optimal conditions: molar ratio Cu(II):TSC:${BH}_{4}^{+}$ = 1:1:0.2, pH = 5, homogenization time 60 min and temperature 25 °C. Thus, the sample was kept in the dark inside a drawer at 25 °C during this process, resulting in the following spectra:

From the resulting spectra, it can be observed that after 24 h there are no major differences in absorbance value. The sample remains stable for 60 days. In order to highlight the monodispersity of the colloidal system, the full width at half maximum values were determined. The acquired results demonstrate that the monodispersity of the CuNPs remains constant, even after a 24 h period. Furthermore, absorbance is steady throughout the investigation.

#### 2.1.7. Proposed Mechanism

The citrate ion [C_6_H_5_O_7_]^3−^ complexes Cu^2+^ in a 1:1 molar ratio according to the equations [81]:

2Cu^2+^ + 2[Cit]^3−^ = [Cu_2_Cit_2_H_−1_]^3−^ stability constant, log β = 10.85 (1)

2Cu^2+^ + 2[Cit]^3−^ = [Cu_2_Cit_2_]^2−^ stability constant, log β = 14.43 (2)

We adopted the stability constants reported by Rode et al. [82], which were mainly based on the work of Daniele et al. [83].

We assume that the mechanism of the reduction process of Cu^2+^ ions is based on the following reactions:

(3) $$2{\mathrm{Cu}}^{2+}(\mathrm{aq})+4{BH}_{4}^{+}(\mathrm{aq})+12H_{2}O=2\mathrm{Cu}(s)+14H_{2}+4B(\mathrm{OH}{)}_{3}$$

(4) $$2{\mathrm{Cu}}^{2+}(\mathrm{aq})+2{BH}_{4}^{+}(\mathrm{aq})+2{\mathrm{OH}}^{-}+5H_{2}O={\mathrm{Cu}}_{2}O+7H_{2}+2B(\mathrm{OH}{)}_{3}$$

It can be assumed that excess ${BH}_{4}^{+}$ is hydrolyzed eventually according to the equation [84]:

(5) $${BH}_{4}^{+}+2H_{2}O={BO}_{2}^{-}+4H_{2}$$

### 2.2. Characterization of Synthesized CuNPs

#### 2.2.1. FT-IR Spectroscopy

**Figure 7:**
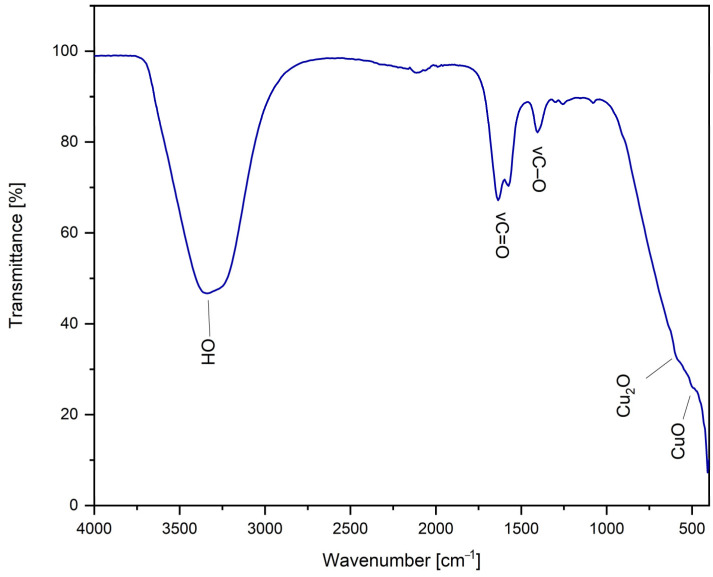
FT-IR spectrum for the sample synthesized under optimal conditions: molar ratio Cu(II):TSC:${BH}_{4}^{+}$ = 1:1:0.2, pH = 5, 60 min homogenization time and 25 °C temperature.

To highlight the functional groups of the synthesized CuNPs, an FT-IR investigation was performed, as shown in Figure 7.

The broad band present at wave number 3343 cm^−1^ is due to the vibration of the O–H group, specific to water [85]. The bands present at wavenumbers of 1634 cm^−1^ and 1407 cm^−1^, respectively, are due to the symmetric and asymmetric vibration of the COO– group specific to TSC [85,86]. The presence of bands at 487 cm^−1^ and 626 cm^−1^ indicates Cu(II)–O stretching vibrations [87].

#### 2.2.2. Raman Spectroscopy

Raman spectroscopy is a versatile technique for characterizing materials at the molecular level, including nanoparticles. In the case of CuNPs, Raman spectroscopy can provide valuable information about the crystal structure, defects and interactions with the environment.

**Figure 8:**
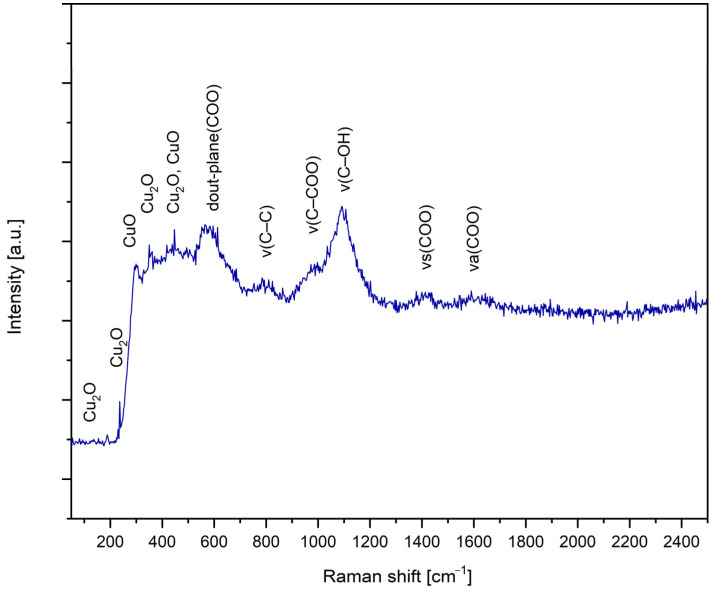
Raman spectrum for the sample synthesized under optimal conditions: molar ratio Cu(II):TSC:${BH}_{4}^{+}$ = 1:1:0.2, pH = 5, 60 min homogenization time and 25 °C temperature.

In Figure 8, the Raman spectrum for CuNPs synthesized under optimal conditions is presented: molar ratio Cu(II):TSC:${BH}_{4}^{+}$ = 1:1:0.2, pH = 5, homogenization time 60 min and temperature 25 °C.

Raman spectra showed several bands specific to Cu_2_O, CuO and Na_3_C_6_H_5_O_7_. The observed bands are located at 108 cm^−1^, 133 cm^−1^, 235 cm^−1^, 291 cm^−1^, 350 cm^−1^, 454 cm^−1^, 591 cm^−1^, 619 cm^−1^, 811 cm^−1^, 859 cm^−1^, 968 cm^−1^, 1098 cm^−1^, 1421 cm^−1^, 1620 cm^−1^ and 2372 cm^−1^.

Most of the important bands are observed for Cu_2_O at wavelengths 108 cm^−1^, 133 cm^−1^, 235 cm^−1^, 291 cm^−1^, 454 cm^−1^ and 591 cm^−1^ [88,89]. The wavelength shift observed for CuNPs can be attributed to the free π electrons provided by TSC [90]. Both the random distribution on the substrate and the stimulation of the various geometric orientations of the CuNP molecules are responsible for the peak improved performance [91].

The presence of CuO bands at ~350 cm^−1^ and 600 cm^−1^ is observed in a small percentage in the material structure [92].

When it comes to the sodium citrate, the specific bands were identified. The 1421 cm^−1^ wavelength corresponds to the carboxylate symmetric stretching band, ν_s_(COO) [93]. Other specific bands were also identified as follows: 1620 cm^−1^ carboxylate asymmetric stretching band ν_a_(COO), 1098 cm^−1^ ν(C–OH), 968 cm^−1^ ν(C–COO), 859 cm^−1^ ν(C–C) and 619 cm^−1^ δ_out-plane_(COO) [93].

#### 2.2.3. Scanning Electron Microscopy Analysis (SEM) and Energy-Dispersive X-Ray Spectroscopy (EDX)

**Figure 9:**
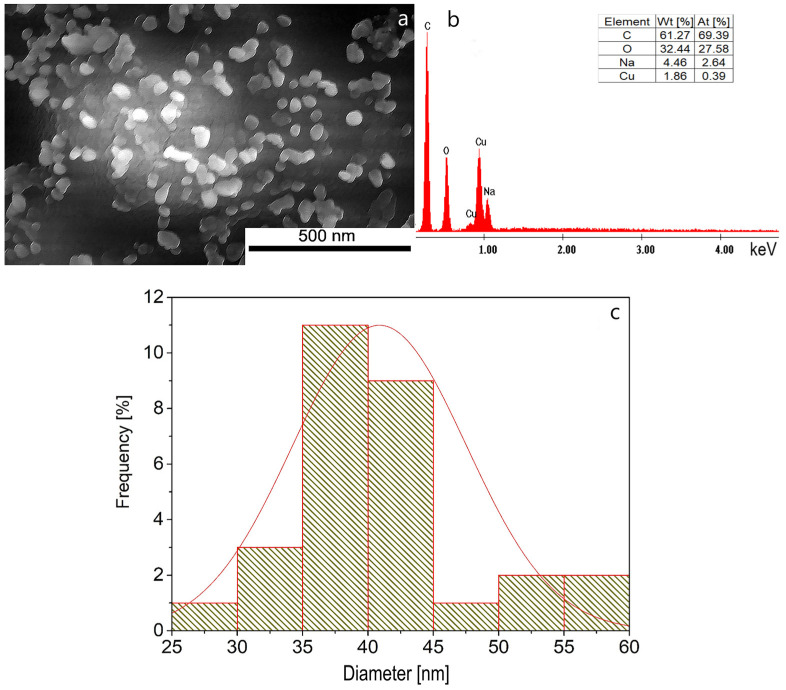
(**a**) Scanning electron microscopy analysis and (**b**) energy-dispersive X-ray spectroscopy of the sample synthesized under optimal conditions: molar ratio Cu(II):TSC:${BH}_{4}^{+}$ = 1:1:0.2, pH = 5, 60 min homogenization time and 25 °C temperature, (**c**) experimental average diameter of CuNPs.

To highlight the surface morphology of the synthesized CuNPs, SEM analysis was performed and is presented in Figure 9. To quantify the elemental composition of the synthesized nanoparticles, EDX spectroscopy was performed.

The morphological and topographical properties of CuNPs were examined by scanning electron microscopy. In addition, the SEM study was used to estimate the average size of CuNPs [77,94]. From SEM microscopy (Figure 9a), it is observed that the obtained CuNPs appear in the form of nanoclusters made up of spherical/quasi-spherical nanoparticles, uniformly dispersed. In the EDX spectrum (Figure 9b), the presence of C, O and Na can be observed, all of which are specific to trisodium citrate, and the presence of Cu confirms the formation of copper particles. Carbon and oxygen peaks in the samples verified the presence of carbon-based stabilizers [95]. The C peak in the spectrum can be attributed to the carbon tape used to mount the sample on the stub. From Figure 9c, it can be seen that the average diameter of CuNPs is 37.5 nm.

Starting from the mass percentages given by the EDX analysis and considering that O comes from the citrate ion and copper oxides that are formed after the reduction, we can state that Cu_2_O is predominant (78%) and that the rest is CuO (22%). This statement is also supported by the Raman spectrum (Figure 8), where Cu_2_O is predominant.

#### 2.2.4. Atomic Force Spectroscopy (AFM)

**Figure 10:**
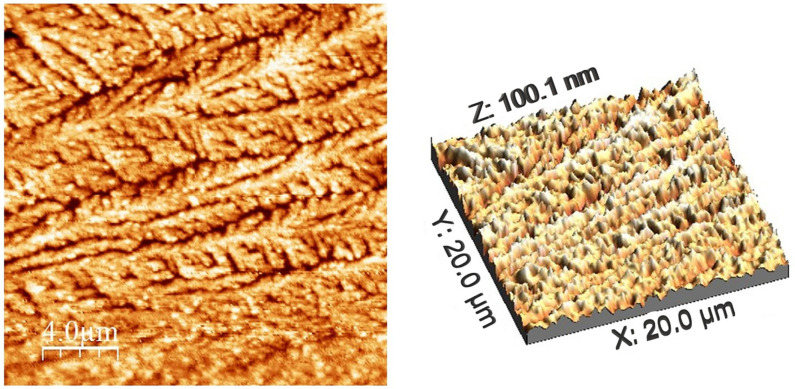
2D and 3D AFM images of the sample synthesized under optimal conditions: molar ratio Cu(II):TSC:${BH}_{4}^{+}$ = 1:1:0.2, pH = 5, 60 min homogenization time and 25 °C temperature.

AFM analysis was performed on a CuNP sample (Figure 10), and roughness data were extracted from the obtained images.

Calculated values from AFM images (average roughness (Sa), mean square root roughness (Sq), maximum peak height (Sp), maximum valley depth (Sv), maximum peak-to-valley height (Sy), surface kurtosis (Sku) and surface skewness (Ssk)) are shown in Table 1:

The material’s registered roughness, as determined by the mean square root roughness (Sq) calculation method, is 20.0095 nm, whereas the average roughness (Sa) calculation formula yields 15.8234 nm. The Sp and Sv values refer to the lowest pits and highest peaks on the analyzed sample. Sy is the sum of the Sp and Sv values, which correspond to the sample’s highest peaks and lowest pits, respectively. Information on abnormally high peaks or deep valleys is provided by the Ssk and Sku values.

**Figure 11:**
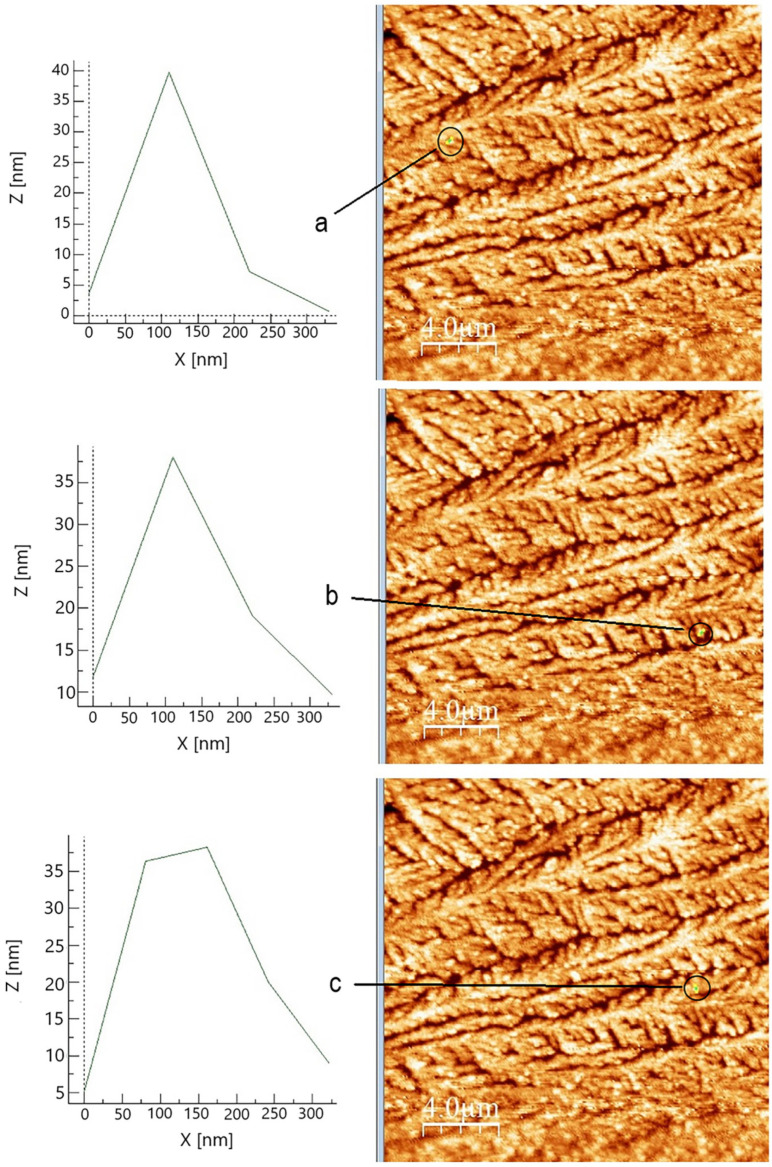
Profile of the selected area of the CuNPs sample synthesized under optimal conditions: molar ratio Cu(II):TSC:${BH}_{4}^{+}$ = 1:1:0.2, pH = 5, 60 min homogenization time and 25 °C temperature in three zone: zone (**a**), zone (**b**) and zone (**c**).

The surface of the acquired CuNP sample is scattered with nanospheres and nano quasi-spheres that resemble the SEM image. The size of the nanoparticles is revealed by the profile analysis of certain regions (Figure 11), which also provides information about the surface features.

However, it should be considered that defects arise during the AFM measurement involving the tip radius curvature effect, resulting in peaks broadening [96]. Therefore, the profile analysis of the spheres is lacking precision, and as result, the calculated size may be erroneous. It is visible that the base of the nanoparticle is always more enlarged in comparison to the tip of the particle. However, the height of the nanoparticles measured in several areas is between 25 and 40 nm, corresponding to the SEM data.

### 2.3. Absorption and Scattering of Electromagnetic Waves by Sphere

To determine the theoretical diameter of CuNPs, a first step involves obtaining a theoretical spectrum “C_ext_/C_sca_/C_abs_ vs. Wavelength”, using the “MiePlot” software for CuNPs. This shows the localized surface plasmon resonance at a wavelength similar to the experimental one, because the localized surface plasmon resonance is strongly influenced by nanoparticle morphology [75]. It is necessary to convert “C_ext_”, obtained from the theoretical spectrum, into the molar absorption coefficient ϵ from the Lambert–Beer law (A = ϵ × l × C), using the following relationship:

(6) $$\epsilon =N_{A}\;\text{×}\;C_{\mathrm{ext}}\;\text{×}\;\ln10\;\text{×}\;(10^{-3}\;\frac{L}{{cm}^{3}})\;[L/mol\times cm]$$

where N_A_ = Avogadro’s number, i.e., N_A_ = 6.022·10^23^ [L/mol], and *C_ext_* = extinction cross section [cm^2^].

In order to theoretically estimate the diameter of spherical CuNPs obtained from the synthesis, the sample with optimal synthesis parameters was used, meaning molar ratio Cu(II):TSC:${BH}_{4}^{+}$ = 1:1:0.2, pH = 5, homogenization time 60 min and temperature 25 °C.

**Figure 12:**
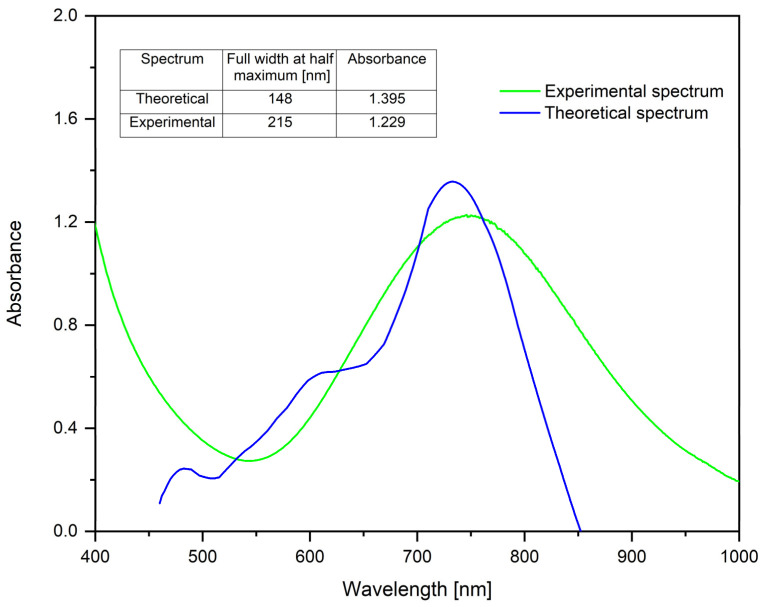
Theoretical determination of CuNP diameter for the sample synthesized under optimal conditions: molar ratio Cu(II):TSC:${BH}_{4}^{+}$ = 1:1:0.2, pH = 5, 60 min homogenization time and 25 °C temperature.

The spectra that are represented in Figure 12 were obtained using the MiePlot software v4.6 [97] and are representative of the synthesized spherical CuNPs particles.

As can be seen, the theoretical spectrum does not perfectly overlap with the experimental one. This may be because the theoretical spectrum was obtained for theoretical nanoparticles with a diameter of ~30 nm, monodisperse. It can also be seen that the full width at half maximum of the experimental spectrum is slightly higher, which implies that the experimentally obtained CuNPs are not exactly monodisperse, the absorbance being lower, so the CuNP concentration is lower compared to the theoretical one.

Based on the calculations, the theoretical size of CuNPs is ~30 nm, whereas the size obtained from the SEM micrograph was 37.5 nm. It can be observed that the theoretical result is relatively similar to that determined by SEM analysis, indicating that this theory is useful in the preliminary determination of the size of spherical/quasi-spherical copper nanoparticles.

### 2.4. CuNP Medical Applications

#### 2.4.1. Microbiologic Tests

The diameter of the inhibitory zone was measured in millimeters to evaluate the antibacterial activity. Following microbiological testing on antibiotic-resistant bacteria, Table 2 displays the determined inhibition diameters on Petri dishes and compares them to the CLSI control test and standards [98].

The values obtained for the diameter disc of inhibition were compared with the interval suggested by the CLSI [98]. The strains used for positive tests are quality control strains, and the interval framing into CLSI measurements demonstrates that the procedure was accurate.

For all strains tested, the inhibition diameter is comparable to that of the CLSI recommendation, which suggests that the tested compound can be associated with an antibiotic in terms of antibacterial activity. At the same time, the tested compound has antibacterial activity against both Gram-negative and Gram-positive strains. This suggests that the tested CuNPs are non-specific and can be used to eliminate a bacterial consortium formed by aerobic bacteria. This is an important aspect in terms of bacterial infections especially, because the predominant aerobic bacteria include *Staphylococcus aureus* and *Escherichia coli* [99], which cause severe infections, and in most cases, patients with malignancy are susceptible to severe infections [100].

The antibacterial activity of CuNPs was compared with that of other metals’ nanoparticles. The comparison is presented in Table 3.

Copper nanoparticles exert an antimicrobial mechanism damaging microbial cells, taking place as a result of reactive oxygen species (ROS) produced through binding or replacing cofactors in metalloproteins [104]. ROS are derivatives which contain unstable oxygen species, such as hydroxyl (OH^•^), hydrogen peroxide (H_2_O_2_) and superoxide ($O_{2}^{*-}$) [105]. Copper can generate ROS through reactions such as [106]:

(7) $${\mathrm{Cu}}^{+}+O_{2}={\mathrm{Cu}}^{2+}+O_{2}^{•-}$$

(8) $$2O_{2}^{•-}+2H^{+}=H_{2}O_{2}+O_{2}$$

Cu^+^ + H_2_O_2_ = Cu^2+^ + OH^•^ + OH^−^ (9)

Copper accepts and donates an electron, jumping between Cu^+^ and Cu^2+^ oxidation states, producing $O_{2}^{•-}$ and the hydroxyl OH^•^ group. These are highly reactive species which lead to lipid peroxidation, protein oxidation and thus DNA damage [107,108].

The fact that CuNPs obtained by the method presented in this study have good antibacterial activity toward both Gram-positive strains tested is a key for its use against infections with Gram-positive strains, because in recent decades, the incidence of infections with Gram-positive bacteria has increased [109].

#### 2.4.2. Cytotoxicity Tests

**Figure 13:**
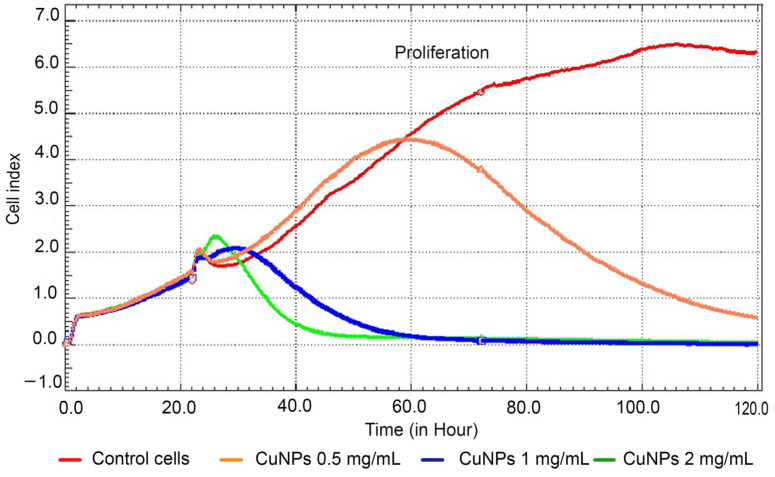
Cytotoxic effects of CuNPs on SKBR3 cells using the xCELLigence system.

A dose-dependent response was observed following exposure to CuNPs synthesized under optimal conditions—namely, molar ratio Cu(II):TSC:${BH}_{4}^{+}$ = 1:1:0.2, pH = 5, homogenization time 60 min and temperature 25 °C—by the SKBR3 cancer cell line. At a concentration of 0.05% (0.5 mg/mL), no significant cytotoxic effects were observed on SKBR3 cells during the initial 60 h, when the cells entered a plateau phase followed by a period of significant decrease in proliferation (Figure 13). However, significant cytotoxicity was evident at concentrations ranging from 0.1% (1 mg/mL) to 0.2% (2 mg/mL) compared to untreated cells (*p* < 0.001), indicating a substantial reduction in cell viability.

The cell index curve illustrates the initial phase of cell adhesion and spreading, followed by a gradual proliferation phase for untreated cells (in red). In contrast, cells treated with CuNPs exhibit a dose-dependent decrease in proliferation (*p* < 0.001). Data are presented as the mean cell index value ± SD.

The study highlights the dose-dependent cytotoxic effects of CuNPs on SKBR3 cancer cells. At low concentrations (0.5 mg/mL), the CuNPs showed minimal impact on cell viability within the first 60 h. However, higher concentrations (1–2 mg/mL) induced pronounced cytotoxicity, indicating their potential as therapeutic agents for cancer treatment. Similar findings were reported by Abdollahzadeh et al., who observed comparable effects in other breast and colorectal cancer cell lines, including MCF-7 and HCT-116 [110].

## 3. Materials and Methods

### 3.1. Synthesis Method

The synthesis of CuNPs is a continuously developing field, and precise control of their size, shape and properties is essential for optimizing the performance of the obtained materials, with specific applications.

To establish the optimal conditions for the synthesis of CuNPs, specific synthesis parameters were established, namely the precursor:reducing agent:complexing agent ratio, the pH of the reaction mass, the homogenizing time and the temperature.

The copper precursor used in this study was copper chloride (Carl Roth, Karlsruhe, Germany), CuCl_2_·2H_2_O 1M. The reducing agent used was sodium borohydride, NaBH_4_ (Merck, Darmstadt, Germany). The complexing agent was trisodium citrate, TSC (Chimexim, Bucharest, Romania), Na_3_C_6_H_5_O_7_ · 3H_2_O 1 M.

**Figure 14:**
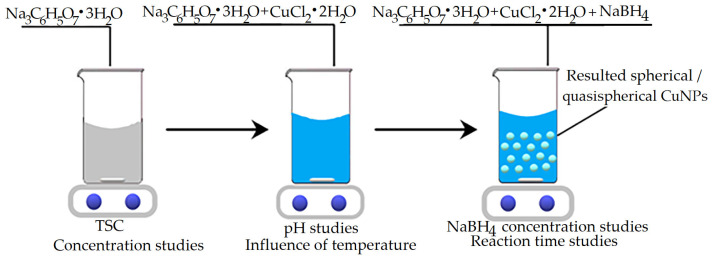
Schematical depiction of CuNP synthesis.

First, 1M TSC was stirred with 1M CuCl_2_ · 2H_2_O for 10 min to homogenize. Then, NaBH_4_ was added to the reaction mixture, followed by homogenization for different time periods (5, 30, 45, 60, 75 and 90 min). The synthesis is schematically presented in Figure 14.

To determine the optimal amount of complexing agent, TSC, the amount was varied so that the molar ratios (Cu(II):TSC:${BH}_{4}^{+}$) would be 1:1:0.2; 1:2:0.2; 1:3:0.2; 1:4:0.2 and 1:5:0.2.

To determine the optimal amount of reducing agent, NaBH_4_, the amounts of precursor and complexing agent were kept constant, varying the amount of reducing agent so as to obtain the molar ratios Cu(II):TSC:${BH}_{4}^{+}$ = 1:1:0.1; 1:1:0.2; 1:1:0.3; 1:1:0.4; 1:1:0.5.

To establish the optimal pH, the pH of the reaction mass was adjusted with a NaOH solution in the range of pH 3–7 using a Mettler Toledo pH meter (Mettler-Toledo International Inc., Columbus, OH, USA). The chosen Cu(II):TSC:${BH}_{4}^{+}$ ratio was the optimal ratio established previously.

The optimum temperature was established by varying the temperature in the range of 10–50 °C by cooling with ice or heating. The reaction mass was set to the optimum Cu(II):TSC:${BH}_{4}^{+}$ ratio.

The stability study of the synthesized CuNPs was performed by UV–Vis analysis at different time intervals (1, 3, 30 and 60 days). During the tests, the samples were kept in the dark, at a temperature of 25 °C.

The produced nanoparticles were evaluated using physicochemical techniques including UV–Vis spectroscopy (Varian Cary 50, Varian Inc., Santa Clara, CA, USA), Raman spectroscopy (at room temperature and 514 nm laser excitation with Shamrock 500i Spectrograph, Angor, UK), FT-IR spectroscopy (Bruker Platinum ATR-QL Diamond spectrometer, Billerica, MA, USA), scanning electron microscopy (SEM) and energy-dispersive X-ray spectroscopy (EDX) (Quanta FEG 250 Scanning, FEI Company, Hillsbro, OR, USA) and atomic force microscopy (AFM) (room-temperature scanning probe microscopy platforms system, with a resonance of 30–40 kHz and a chromium-doped tip with a radius of 20 nm, MultiView-2000, Nanonics Imaging Ltd., Jerusalem, Israel) in order to gain a better knowledge of the structure and characteristics of CuNPs.

In physical optics, the scattering of light by spherical particles is described mathematically by Mie theory. In this study, Mie theory was used to theoretically assess the size of spherical CuNPs. Because it makes it possible to calculate the interaction between light and particles of sizes similar to the incident light’s wavelength, this theory is crucial for characterizing nanoparticles. The MiePlot v4.6 software includes a database which is used to carry out the required computations for the produced copper nanospheres.

### 3.2. Microbiological Tests

#### 3.2.1. Inoculum Preparation

A sterile cotton swab was used to pick colonies from a 24 h culture on Columbia agar + 5% sheep blood (Oxoid, Germany). The picked colonies were transferred to 0.9% saline, and an inoculum was obtained. The inoculum density was adjusted to 0.5 McFarland (1–2 × 10^8^ microorganisms/mL) by addition of bacterial colonies or saline solution. The standardized inoculum was used within 15 min of preparation.

#### 3.2.2. Inoculation of Agar Plates

A sterile cotton swab was used to take the inoculum from the 0.5-McFarland-density suspension of the following bacterial strains: Gram-negative *Escherichia coli* ATCC 25922, and Gram-positive *Staphylococcus aureus* ATCC 29213 and *Streptococcus pneumoniae* ATCC 49619 (Microbiologics, Couëron, France). Mueller–Hinton agar (Oxoid, Germany) was inoculated with 0.1 mL of this standardized suspension, which was distributed uniformly over the entire surface of the agar. To test the antibacterial effect on *Streptococcus pneumoniae*, Mueller–Hinton medium supplemented with sheep blood (Oxoid, Germany) was used.

Of the compound to be tested, 10 µL was added to each empty disc (BioMaxima, Lublin, Poland), which, after impregnation, was dried in a sterile environment. Each disc with the test material was subsequently placed in the center, on the surface of the medium, for each microbial strain tested, while other antibiotic microtablets were placed around it and used as controls for the interpretation of the antibacterial effect.

#### 3.2.3. Application of Antimicrobial Discs

Positive controls used for testing were represented by 2 antibiotics for each strain tested (Oxoid, Germany). For the *E. coli* strain, 10 µg gentamicin and 30 µg cefepime were used; for *S. aureus* testing, 30 µg cefoxitin and 10 µg gentamicin were used; and for *S. pneumoniae*, 15 µg erythromycin and 30 µg tetracycline were used.

A disc impregnated with 10 µL of the test compound was also applied, to test the sensitivity to this compound.

#### 3.2.4. Principle of the Diffusimetric Method

The plates inoculated with the microbial suspensions were incubated at 35 ± 2 °C for 24 h.

The presence of sensitivity to the antibiotics used was considered a positive control.

The diameters of the inhibition zones obtained (which include the diameter of the disc) were measured in mm using a ruler. The reading of the inhibition zone limit was assessed with the naked eye, with the plate held approximately 30 cm from the eye.

### 3.3. Biological Test

#### Cell Culture and Treatment

The cells were maintained in McCoy’s 5A medium (Gibco, Life Technologies, Grand Island, NY, USA) containing 10% FBS (PromoCell, Heidelberg, Germany) and 1% penicillin–streptomycin (Gibco, Life Technologies, Grand Island, NY, USA) in a CO_2_ incubator at 37 °C. Cell toxicity of CuNPs was assessed on the human breast cancer cell line SKBR3 (catalog number HTB-30, American Type Culture Collection, Virginia, USA) using the xCELLigence system (ACEA Biosciences, San Diego, CA, USA). This system enables real-time, non-invasive monitoring of adherent cell proliferation and viability through electronic impedance measurement. For these measurements, cells were seeded in 200 µL of culture medium in specialized microplates (E-Plate, ACEA Biosciences, San Diego, CA, USA) at nanoparticle concentrations of 0.05% (0.5 mg/mL), 0.1% (1 mg/mL) and 0.2% (2 mg/mL). The system continuously monitored cell attachment, spreading and proliferation. Twenty-four hours after seeding, nanoparticle treatments were applied, and cell behavior was tracked for up to 120 h. Cell impedance was expressed as the cell index, a parameter automatically calculated by the system’s software (xCelligence, RTCA Software 1.2, version 1.2.1). The cell index is a relative measurement, a unitless parameter that is automatically determined by dividing the change in electrical impedance (R) at a specific frequency by the nominal impedance value [111]. This method provided precise and dynamic insights into cell responses to CuNPs.

The percentage of cytotoxicity (%) in cells exposed to CuNPs at different concentrations was calculated relative to the control values based on the cell index [112], following the formula:

(10) $$\mathrm{Cytotoxicity}(\%)=(1-\frac{cell\;index\;of\;treated\;group\;at\;time\;t\;}{cell\;index\;of\;control\;group\;at\;time\;t\;})\;\times \;100$$

The results are presented as mean value ± standard deviation. A one-way ANOVA test was used to determine the statistical difference between the experimental groups.

## 4. Conclusions

The present study illustrates a simple, convenient and significant method for the synthesis of CuNPs.

The study demonstrated the possibility of synthesizing CuNPs by chemical reduction using NaBH4 as a reducing agent and TSC as a complexing agent. The established optimal synthesis conditions are: molar ratio Cu(II):TSC:${BH}_{4}^{+}$ = 1:1:0.2, pH = 5, homogenization time 60 min and temperature 25 °C. The synthesized CuNPs were characterized by physicochemical characterization using UV–Vis spectroscopy. At λ = ~746 nm, the presence of CuNPs was revealed. SEM micrographs and EDX spectra indicated that the synthesized CuNPs are spherical/quasi-spherical in shape, with a diameter of ~37 nm. The particle size was also confirmed by AFM investigation.

FT-IR spectroscopy highlighted the presence of vibrational bands for Cu–O. Using Raman spectroscopy, the presence of a mixture of Cu_2_O and CuO particles was identified. The sample remained stable for 60 days. In order to highlight the monodispersity of the colloidal system, it was determined that the theoretical diameter of CuNPs involves obtaining a theoretical spectrum “C*_ext_*/C*_sca_*/C*_abs_* vs. Wavelength”, using the “MiePlot” software v4.6 for CuNPs, which was compared with the experimentally obtained spectrum. Based on the calculations, the theoretical size of CuNPs was ~30 nm, whereas the size obtained from the SEM micrograph was 37.5 nm. Thus, we can conclude that the theoretical result is relatively similar to that determined by SEM analysis, indicating that Mie theory is useful in the determination of the size of spherical/quasi-spherical nanoparticles, in general.

CuNPs reveal very good antibacterial activity, on both Gram-negative and Gram-positive bacteria. Their antibacterial activity is comparable to the CLSI standard recommendation for all the tested strains. This suggests that CuNPs can be further investigated for medical application in terms of bacterial infection eradication.

The capacity of metallic nanoparticles to overcome antibiotic resistance has been attributed to their special physicochemical characteristics, which allow for the exploitation of several new bactericidal routes in order to provide antimicrobial activity. Metallic nanoparticles have a wide range of activity because of the typically non-specific binding between metal ions and the biomolecules of microorganisms.

This study suggests that CuNPs exhibit dose-dependent cytotoxicity on the SKBR3 cancer cell line. However, the increase in cytotoxic effect requires further investigation, particularly with normal cell lines, to assess the potential of CuNPs as therapeutic agents for cancer treatment. Additional research is essential to elucidate the underlying mechanisms driving cytotoxicity and optimize these CuNPs for their use in therapeutic applications.

## Figures and Tables

**Table 1:** Values obtained from AFM analysis.

Sample Name	Ironed Area (µm^2^)	Sa (nm)	Sq (nm)	Sp (nm)	Sv (nm)	Sy (nm)	Sku	Ssk
CuNPs	409.886	15.8234	20.0095	46.1396	−53.9583	100.097	2.8463	−0.3843

**Table 2:** The inhibition diameter for reference strains tested.

ATCC Strains	Tested Compound 10 µL [mm]	Positive Control Test [mm]	Standard CLSI [mm]	Antibiotics Used in Positive Control Test
*Streptococcus pneumoniae* ATCC 49619	22	30	≥21	ERYTHROMICIN 15 µg
		30	≥23	TETRACICLINE 30 µg
*Staphylococcus aureus* ATCC 25923	22	29	≥22	CEFOXITIN 30 µg
		26	≥15	GENTAMICIN 10 µg
*Escherichia coli* ATCC 25922	21	35	≥18	CEFEPIME 30 µg
		24	≥15	GENTAMICIN 10 µg

**Table 3:** Antibacterial activity of CuNPs compared with other metallic nanoparticles.

Metallic Nanoparticles Tested	ATCC Strains	Diameter of Inhibition [mm]	References
CuNPs, 10 µL	*Streptococcus pneumoniae* ATCC 49619	22	Present paper
	*Staphylococcus aureus* ATCC 25923	22	
	*Escherichia coli* ATCC 25922	21	
AgNPs, 20 µL	*Staphylococcus aureus* ATCC 25923	19	[101]
	*Pseudomonas aeruginosa* ATCC 27853	16	
	*Escherichia coli* ATCC 25922	16	
	*Streptococcus pneumoniae* ATCC 49619	11	
	*Candida albicans* ATCC 90028	20	
AgNPs, 10 µL	*Escherichia coli* ATCCT 8739	8.9	[102]
	*Pseudomonas aeruginosa* ATCCT 27853	9.7	
	*Staphylococcus aureus* ATCC 25923	10.7	
	*Salmonella enterica* NCTC 160	12.2	
	*Bacilus cereus* ATCC 14579	10.4	
CuNPs 50 µL	*Escherichia coli* MTCC 4296	13.00	[103]
	*Pseudomonas aeruginosa* MTCC 424	16.00	
	*Bacillus cereus* MTCC 619	12.17	
	*Staphylococcus aureus* MTCC 316	9.67	

## Data Availability

Data are contained within the article.
